# Risk factors for neurocognitive impairment in HIV-infected patients and comparison of different screening tools

**DOI:** 10.1590/S1980-57642016DN10100008

**Published:** 2016

**Authors:** Elisa Moreira de Souza, Caroline Schleiffer Buoniconti, Frederico Cunha Valim, Alexandre Sampaio Moura

**Affiliations:** 1 Estudante de Gradução. Curso de Medicina, Universidade José do Rosário Vellano, Belo Horizonte MG, Brasil; 2 Doutor Professor de Curso de Medicina, Universidade José do Rosário Vellano, Belo Horizonte MG, Brasil

**Keywords:** HIV, dementia, Mini-Mental State Examination, International HIV Dementia Scale, HIV, demência, Mini Exame do Estado Mental, Escala Internacional de Demência pelo HIV

## Abstract

HIV-associated neurocognitive disorder (HAND) is relatively frequent among HIV-infected patients and is often underdiagnosed. Assessment of HAND in daily clinical practice is challenging and different tools have been proposed.

**Objective:**

To evaluate risk factors and compare different screening tools for neurocognitive impairment in HIV-infected patients.

**Methods:**

HIV-infected patients were evaluated using the International HIV-Dementia Scale (IHDS), Mini-Mental State Examination (MMSE) and a neurocognitive self-perception questionnaire recommended by the European AIDS Clinical Society. Sociodemographic, clinical and laboratory data were obtained through chart review and patient interview.

**Results:**

Among the 63 patients included, low performance on the IHDS was observed in 54.0% and IHDS score was inversely associated with age (OR 0.13; 95%CI [0.02-0.67]). Regarding cognitive self-perception, 63.5% of patients reported no impairment on the three domains covered by the questionnaire. Among those patients self-reporting no problems, 42.1% had low performance on the IHDS. None of the patients scored below the education-adjusted cut-off on the MMSE.

**Conclusion:**

IHDS scores suggestive of HAND were observed in more than half of the patients and lower scores were found among older patients. There was low agreement between the different tools, suggesting that the MMSE may be inadequate for assessing HAND. The self-assessment questionnaire had low sensitivity and might not be useful as a screening tool.

## RESUMO

As alterações neurocognitivas associadas ao HIV (HAND) são relativamente frequentes entre pacientes infectados pelo HIV, porém são subdiagnosticadas. Avaliação de HAND na prática clínica diária é desafiador e diferentes ferramentas têm sido propostas.

**Objetivo:**

Avaliar fatores de risco e comparar diferentes ferramentas de rastreamento de alterações neurocognitivas em pacientes infectados pelo HIV.

**Métodos:**

Pacientes infectados pelo HIV foram avaliados usando a Escala Internacional de Demência pelo HIV (IHDS), Mini Exame do Estado Mental (MEEM) e um questionário de autopercepção neurocognitiva recomendado pela Sociedade Clínica Europeia de AIDS. Dados sociodemográficos, clínicos e laboratoriais foram obtidos por revisão de prontuário e entrevista com o paciente.

**Resultados:**

Entre os 63 pacientes incluídos no estudo, um baixo desempenho no IHDS foi observado em 54,0% e o escore no IHDS esteve inversamente associado à idade (OR 0,13; IC95% [0,02-0,67]). Em relação à autopercepção cognitiva, 63,5% dos pacientes não relataram nenhum prejuízo nos três domínios avaliados pelo instrumento. Nenhum paciente apresentou escore no MEEM abaixo do ponto de corte ajustado para escolaridade.

**Conclusão:**

Escores no IHDS sugestivos de HAND foram observados em mais da metade dos pacientes e valores mais baixos foram encontrados entre pacientes mais velhos. Houve pouca concordância entre os diferentes métodos de avaliação, sugerindo que o MEEM é inadequado para avaliação de HAND e o questionário de auto-avaliação tem uma baixa sensibilidade, não parecendo ser útil como ferramenta de triagem.

## INTRODUCTION

The central nervous system (CNS) is a major target for HIV infection with high viral loads often observed in cerebrospinal fluid and in different anatomical sites such as the caudate nucleus and the hippocampus of HIV-infected patients.^1^ In the CNS, HIV infects and replicates on macrophages, microglia and multicellular glia, resulting in the release of neurotoxic factors and subsequent cell damage.^2^

In 1991, the American Academy of Neurology divided HIV-associated neurocognitive disease (HAND) into two different categories: HIV-associated dementia (HAD) and mild neurocognitive disorder. HAD is characterized by impairment in multiple domains, particularly learning of new information, information processing, and attention or concentration, that impact at least two activities of daily living and result in at least one functional or psychosocial change. In mild neurocognitive disorder, there is a reduction in mental accuracy with loss of efficiency at work and reduced performance on domestic tasks,^1^ but with a much lower impact on activities of daily living.

A decade later, Antinori et al.,^3^ introduced a new category of asymptomatic neurocognitive disorders based on the finding that some individuals have subclinical impairment on neurocognitive evaluation without any impact on activities of daily living. Prevalences of asymptomatic neurocognitive disorder (ANA), mild neurocognitive disorder and HAD are 30-35%, 20-25% and 2-3%, respectively.^4^ CD4+ cell counts lower than 200 cells/mm³, age greater than 50 years and low educational status were found to be risk factors for HIV-associated neurocognitive disorders (HAND).^4^

The gold standard for diagnosing neurocognitive impairment is a battery of neuropsychological tests applied by a trained neuropsychologist. However, such comprehensive assessment is not feasible in daily clinical practice and simpler screening tools are needed.^4^^,^ 5

The IHDS is a rapid assessment tool that evaluates memory-recall and both motor and psychomotor speed.^5^^,^ 6 It consists of three subtests: [1] timed finger tapping which measures motor speed; [2] timed alternating hand sequence test which assesses psychomotor speed; and [3] recall of 4 words (blue, dog, hat and apple) at 2 minutes which assesses memory registration and recall. Each of these tests is rated on a scale of 0-4 and the maximum possible score on the IHDS is 12. Validation of the IHDS in Brazil was conducted by Rodrigues et al.,^5^ and showed sensitivity and specificity for detecting HAND of 55% and 80%, respectively. A moderate-to-high interobserver agreement was observed and a there was reasonable agreement between the IHDS and other neuropsychological tests.^5^

Another neurocognitive assessment tool is the Mini-Mental State Examination (MMSE) that evaluates orientation, attention and calculation, registration, recall, language and the ability to follow simple commands.^7^ However, the MMSE was originally developed to screen for cortical dementia such as Alzheimer's disease and there might be limitations on its use to assess subcortical disorders, such as those observed among HIV-infected patients.^7^

A self-perception questionnaire was recently proposed by the European AIDS Clinical Society as a first step in neurocognitive evaluation of HIV-infected patients.^9^ The questionnaire has three items related to memory, attention and information processing, based on a previous study conducted by Simioni et al.^8^ However, the performance of the questionnaire in clinical setting has not been systematically evaluated.

The aim of this study was to evaluate the factors associated with performance on the IHDS and MMSE and level of agreement between scores on these screening tools and patients' self-perception of neurocognitive status.

## METHODS

Between October 2013 and February 2014, consecutive HIV-infected patients were recruited for the study at CEASC-UNIFENAS, a public university-based ambulatory-care unit for infectious diseases located in Belo Horizonte, Brazil. All patients included were adults and had confirmed HIV diagnosis. Patients were excluded if they were illiterate, had severe psychiatric conditions or current CNS opportunistic infection. Sociodemographic, clinical and laboratory data were obtained through chart review and patient interview. 

Neurocognitive evaluation using validated versions of the IHDS^5^ and MMSE^7^ was conducted by trained researchers. For the IHDS, a score less than or equal to 10 was considered to be altered, based on the study of Rodrigues et al. that showed a sensitivity and specificity for detection of HAND of 55% and 80%, respectively.^5^ For the MMSE, a cut-off score based on years of education was used as following: 18 points for patients with four years of education or less and 26 points for those with more than four years of education.^7^

Cognitive self-perception was assessed by a questionnaire recommended by the European AIDS Clinical Society guideline.^8^^,^ 9 The questionnaire includes three items: [1] "Do you experience frequent memory loss?"; [2] "Do you feel that you are slower when reasoning, planning activities or solving problems?"; [3] "Do you have difficulties paying attention?". For each of the questions, patients must choose one of the following answers: [a] never, [b] hardly ever or [c] yes, definitely. The EACS guideline recommends that patients be submitted to a more thorough neurocognitive evaluation if the response on at least one of the items is "yes, definitely". 

Patients' depressive symptoms were assessed using the Beck Depression Inventory (BDI),^10^ a self-rated 21-item questionnaire validated in Brazil by Gorenstein and Andrade.^11^ A score of < 14 suggests the presence of no or minimal depressive symptoms while scores from 14-19, 20-28 and 29-63 are suggestive of the presence of mild, moderate or severe depressive symptoms, respectively.

Demographic information, CD4+ cell count, HIV viral load, antiretroviral regimen, smoking history, use of illicit drugs and alcohol abuse were obtained from patients' medical records. 

Descriptive analysis of frequency and proportions were used for categorical variables. Comparison of proportions was conducted using Pearson's Chi-square test. Means and standard deviation were used for normally distributed continuous variables. Statistical significance was set up at 0.05. The Epi info statistical package (Version 3.5.4, July 30 (2012)) was used to conduct all analyses. 

This study was conducted in accordance with the Helsinki declaration and approved by the local Research Ethics Committee. All subjects gave their written informed consent.

## RESULTS

Sixty-four patients were assessed throughout the study period and one was excluded due to illiteracy. Among the 63 patients included, 45 (71.4%) were male, with a mean age of 42.9 years (range 19.0-73.0), 39 (61.9%) individuals were non-white and 38 (60.3%) had eight or less years of education. Only two patients (3.2%) had a CD4+ count less than 200cells/mm³. Fifty-eight patients were on antiretroviral therapy (ART) and 30 (51.7%) of these were using efavirenz. Among the forty-four patients that had been on ART for more than 24 weeks, 37 (84.1%) had undetectable viral load. 

Twenty-four (33.9%) patients showed symptoms of depression, where most of these were suggestive of mild depression. Only nine patients had a BDI score suggestive of moderate or severe depression (Table 1). 

**Table 1.** Baseline demographic and clinical characteristics of all patients included.

	**N=63**
Age (years)	42.9 ± 10.9*
Years of education (%)	
1-8	60.3%
9-12	30.1%
>12	9.5%
Male (%)	71.4%
Non-white (%)	61.9%
CD4+ T-cell count < 200 /mm^3^ (%)	3.2%
CD4+ T-cell count (cells/mm^3^)	556.6 ± 189.1*
Viral load <50 copies/ml (%)	58.7%
Current ARV use	92.0%
Efavirenz use	51.7%
Beck Depression Inventory score (%)	
0-13 points	66.1%
14-19 points	25.4%
20-28 points	8.5%
>29 points	6.3%

*mean ± standard deviation.

Neurocognitive assessment showed that 34 (54.0%) patients had low performance (<11) on the IHDS (Table 2) and scores were inversely associated with age (OR 0.13; 95%CI 0.02-0.67). Performance on the IHDS was not significantly associated with efavirenz use, gender, CD4+ cell count; viral load or depressive symptoms (Table 3).

**Table 2.** Performance of HIV-infected patients on different neurocognitive assessment tools.

	**N=63**
IHDS	
≥ 11	36.0%
< 11	54.0%
MMSE	
≥ cut-off point*	100% 0%
< cut-off point*	0%
EACS self-perception questionnaire	
Reported no problems Reported at least one problem	74.3%
Reported at least one problem	25.7%

EACS: European AIDS Clinical Society; IHDS: International HIV Dementia Scale; MMSE: Mini-Mental State Examination; *Adjusted by educational level

**Table 3.** Univariate analysis of factors associated with performance on the International HIV Dementia Scale (IHDS).

	**IHDS≤10 (n=34)**	**IHDS>10 (n=29)**	**OR**	**95% CI**	**P value**
Age ≤ 50 (years)	22 (64.7%)	27 (93.0%)	0.13	0.02-0.67	< 0.01
Efavirenz use	18 (54.9%)	12 (41.3%)	1.73	0.61-4.90	0.30
CD4 count < 200 cell/mm^3^	1 (2.9%)	1 (3.4%)	0.84	0.05-14.19	0.90
Viral load ≥ 50 copies/ml	14 (41.1%)	12 (41.3%)	1.0	0.36-2.75	0.49
BDI score < 13	20 (58.8%)	19 (65.5%)	0.75	0.26-2.09	0.66

BDI: Beck Depression Inventory.

None of the patients included had an MMSE score below the cut-off level. 

Regarding the self-assessment questionnaire, 25.7% of the patients answered positively for at least one of the questions (Table 2). Among those patients self-reporting no problems, 42.1% had low performance on the IHDS.

## DISCUSSION

A high proportion of HIV-infected patients were found to have impaired performance on the IHDS. A similar high prevalence of HAND, as suggested by low scores on the IHDS, was also reported by Oshinaike et al. in Nigeria^12^ and Rodrigues et al. in Brazil.^5^

With the introduction of highly-active antiretroviral therapy (HAART), a reduction in the incidence of HAND was observed but its prevalence has increased due to improved patient survival.^5^^,^ 10 Currently, as the HIV population is becoming older, both incidence and prevalence of HAND appear to be increasing.^13^ Indeed, we have found age to be associated with performance on the IHDS - a finding also reported by others.^14^^,^ 15

We failed to find an association between CD4+ cell count and performance on the IHDS, such as the associations found by Antinori et al.^3^ However, the number of patients with CD4+ cell count less than 200 cells/mm^3^ in the present study was too small to make meaningful comparisons. 

Efavirenz is associated with a variety of psychiatric and neurological conditions due to its neurotoxicity.^16^ However, we found no association between efavirenz use and performance on the IHDS, in agreement with the results of Lopardo et al.^15^

Depression is frequent among HIV-infected patients. Kagee & Martin conducted a study in South Africa using BDI and estimated a prevalence of moderate and severe depression of 37.4% and 20%, respectively.^17^ Our prevalence of depressive symptoms was much lower and was not associated with IHDS performance.

None of the patients in the present study showed alterations on the MMSE. This finding reinforces the claim made by Sacktor et al.^6^ that the MMSE is useful for cortical dementias but might lack sensitivity when evaluating subcortical neurological disorders such as those associated with HIV.

Regarding the self-assessment neurocognitive questionnaire recommended by the EACS,^8^ we have found an unacceptable high rate of patients without complaints (42.1%) that had impaired performance on the IHDS, thereby limiting its utility as a screening tool. Simioni et al.,^8^ (also reported a high prevalence of HAND among HIV-infected patients with long-standing undetectable viral load without neurocognitive complaints.

Misdiagnosis of HAND can have a significant impact on HIV care. HAND overdiagnosis might reduce patients' self-esteem, lead to inappropriate medical interventions and increases the already high cost of AIDS treatment.^18^ Conversely, lack of early diagnosis might delay appropriate interventions such as antiretroviral therapy modifications.^19^

The present study has several limitations. First, patients were recruited from a single referral center and might not reflect the overall HIV-infected population. In addition, the small sample size may have limited the power for identifying some risk factors for HAND such as CD4 cell count. Finally, the study did not include a thorough neuropsychological assessment. However, the IHDS has been previously shown to have good performance compared to more comprehensive neurocognitive assessment.^5^

In summary, a high proportion of HIV-infected patients had poor performance on the IHDS, suggestive of HAND, a trait that seemed to increase with age. The self-assessment questionnaire recommended by the EACS and the MMSE might have a limited role as screening tools for neurocognitive impairment in HIV-infected patients.
